# The Fc-region of a new class of intact bispecific antibody mediates activation of accessory cells and NK cells and induces direct phagocytosis of tumour cells

**DOI:** 10.1054/bjoc.2000.1237

**Published:** 2000-06-15

**Authors:** R Zeidler, J Mysliwietz, M Csánady, A Walz, I Ziegler, B Schmitt, B Wollenberg, H Lindhofer

**Affiliations:** Clinical Cooperation Group ‘Bispecific Antibodies’ at the Department of Otorhinolaryngology, Ludwig-Maximilians-University, Marchioninistrasse 15, Munich, 81377; Institutes of Molecular Immunology, GSF-National Research Center for Environment and Health, Marchioninistrasse 25, Munich, 81377, Germany; Clinical Molecular Biology, GSF-National Research Center for Environment and Health, Marchioninistrasse 25, Munich, 81377, Germany

**Keywords:** phagocytosis, accessory cells, bispecific antibody, tumour

## Abstract

Bispecific antibodies (bsAb) are considered as promising tools for the elimination of disseminated tumour cells in a minimal residual disease situation. The bsAb-mediated recruitment of an immune effector cell in close vicinity of a tumour cell is thought to induce an antitumoural immune response. However, classical bispecific molecules activate only a single class of immune effector cell that may not yield optimal immune responses. We therefore constructed an intact bispecific antibody, BiUII (anti-CD3 × anti-EpCAM), that not only recognizes tumour cells and T lymphocytes with its two binding arms, but also binds and activates Fcγ-receptor positive accessory cells through its Fc-region. We have demonstrated recently that activated accessory cells contribute to the bsAb-induced antitumoural activity. We now analyse this stimulation in more detail and demonstrate here the BiUll-induced upregulation of activation markers like CD83 and CD95 on accessory cells and the induction of neopterin and biopterin synthesis. Experiments with pure cell subpopulations revealed binding of BiUll to CD64+ accessory cells and CD16+ NK cells, but not to CD32+ B lymphocytes. We provide further evidence for the importance of the Fc-region in that this bispecific molecule stimulates Fcγ-R-positive accessory cells to eliminate tumour cells in vitro by direct phagocytosis. © 2000 Cancer Research Campaign


*British Journal of Cancer* (2000) **83**(2), 261–266
© 2000 Cancer Research Campaign
doi: 10.1054/ bjoc.2000.1237, available online at http://www.idealibrary.com on IDEAL®

### The Fc-region of a new class of intact bispecific antibody mediates activation of accessory cells and NK cells and induces direct phagocytosis of tumour cells

**R Zeidler[1], J Mysliwietz[2], M Csánady[1], A Walz[1], I Ziegler[3], B Schmitt[1], B Wollenberg[1] and H Lindhofer[1]**

[1]Clinical Cooperation Group 'Bispecific Antibodies' at the Department of Otorhinolaryngology, Ludwig-Maximilians-University, Marchioninistrasse 15, 81377 Munich; [2]Institutes of Molecular Immunology and [3]Clinical Molecular Biology, GSF-National Research Center for Environment and Health, Marchioninistrasse 25, 81377 Munich, Germany

**Summary** Bispecific antibodies (bsAb) are considered as promising tools for the elimination of disseminated tumour cells in a minimal residual disease situation. The bsAb-mediated recruitment of an immune effector cell in close vicinity of a tumour cell is thought to induce an antitumoural immune response. However, classical bispecific molecules activate only a single class of immune effector cell that may not yield optimal immune responses. We therefore constructed an intact bispecific antibody, BiUII (anti-CD3 × anti-EpCAM), that not only recognizes tumour cells and T lymphocytes with its two binding arms, but also binds and activates Fcγ-receptor positive accessory cells through its Fc-region. We have demonstrated recently that activated accessory cells contribute to the bsAb-induced antitumoural activity. We now analyse this stimulation in more detail and demonstrate here the BiUll-induced upregulation of activation markers like CD83 and CD95 on accessory cells and the induction of neopterin and biopterin synthesis. Experiments with pure cell subpopulations revealed binding of BiUll to CD64+ accessory cells and CD16+ NK cells, but not to CD32+ B lymphocytes. We provide further evidence for the importance of the Fc-region in that this bispecific molecule stimulates Fcγ-R-positive accessory cells to eliminate tumour cells in vitro by direct phagocytosis.
© 2000 Cancer Research Campaign

**Keywords:** phagocytosis; accessory cells; bispecific antibody; tumour

Bispecific antibodies are regarded as efficient tools for the immunological treatment of disseminated tumour cells in minimal residual disease situations. Usually, they are constructed to target tumour cells by a specific or tumour-associated antigen and to recruit one class of immune effector cell, either T cells or accessory cells like monocytes or natural killer cells. However, long-lasting immune reactions in vivo are much more complex and depend on the activation of different classes of immune effector cells, especially in the initial phase of the immune response. This is usually regarded as the major drawback of conventional bsAb that may not yield full immune responses at the tumour site. We have developed a new class of bispecific antibody, that is composed of the two potent subclasses mouse IgG2a × rat IgG2b. BiUII, a member of these new bispecific molecules, targets tumour cells via the pan-carcinoma antigen EpCAM and T-lymphocytes via CD3. But, in contrast to other bispecific molecules described to date (Fanger et al, 1990; Valerius et al, 1997; Weiner et al, 1995), it also binds and activates human Fcγ-receptor-positive accessory cells like monocytes/macrophages, NK cells, and dendritic cells (DCs) via its Fc-region. Activation of these accessory cells results in the upregulation of costimulatory molecules like CD40, CD80, and CD86 and the production of cytokines like IL-2, IL-6, and DC-CK1 (Zeidler et al, 1999).

Although T cells are considered to be the most important cells for tumour cell elimination, they depend on proper antigen presentation by professional antigen-presenting cells (APCs) or activated accessory cells and costimulatory molecules like CD40, LFA-3, CD80, and CD86 in the presence of cytokines such as IL-2 and IL-12 (Inaba and Steinman, 1984; Stüber et al, 1996). This reveals the importance of the subclass combination for induction of activation signals via the Fc-receptor of accessory cells. A similar T-cell redirecting bsAb, SHR-1 (anti-CD3 × anti-CD19), with the subclass combination mouse IgG1 × rat IgG2b was neither able to activate accessory cells via its Fc-region in a clinical study (de Gast et al, 1995) nor in in vitro assays without addition of exogenous IL-2 (Klein et al, 1997). Moreover, the antitumour efficiency of BiUII is strongly reduced when T cells alone are used as effector cells. We therefore postulate that only the activation of more than one class of immune effector cell is necessary to provide optimal antitumour efficiency. Furthermore, phagocytosis, processing, and presentation of tumour material by APCs are prerequisites for the induction of a polyclonal humoral and cellular antitumour immune response. These data are in accordance with the work of Clynes et al (1998), who recently demonstrated the importance of Fc receptors in passive and active immunity to a melanoma model.

*Received 7 December 1999*
*Revised 2 March 2000*
*Accepted 10 March 2000*

*Correspondence to:* H Lindhofer

### MATERIALS AND METHODS

### Cell lines and PBMC preparation

PCl-I (a gift from Dr T Whiteside, Pittsburgh, PA, USA) is an adherent squamous carcinoma cell-line of the head and neck (SCCHN) and is kept in DMEM with 10% FCS. The cell-line expresses EpCAM but lacks CD80 and CD86 as tested by flow

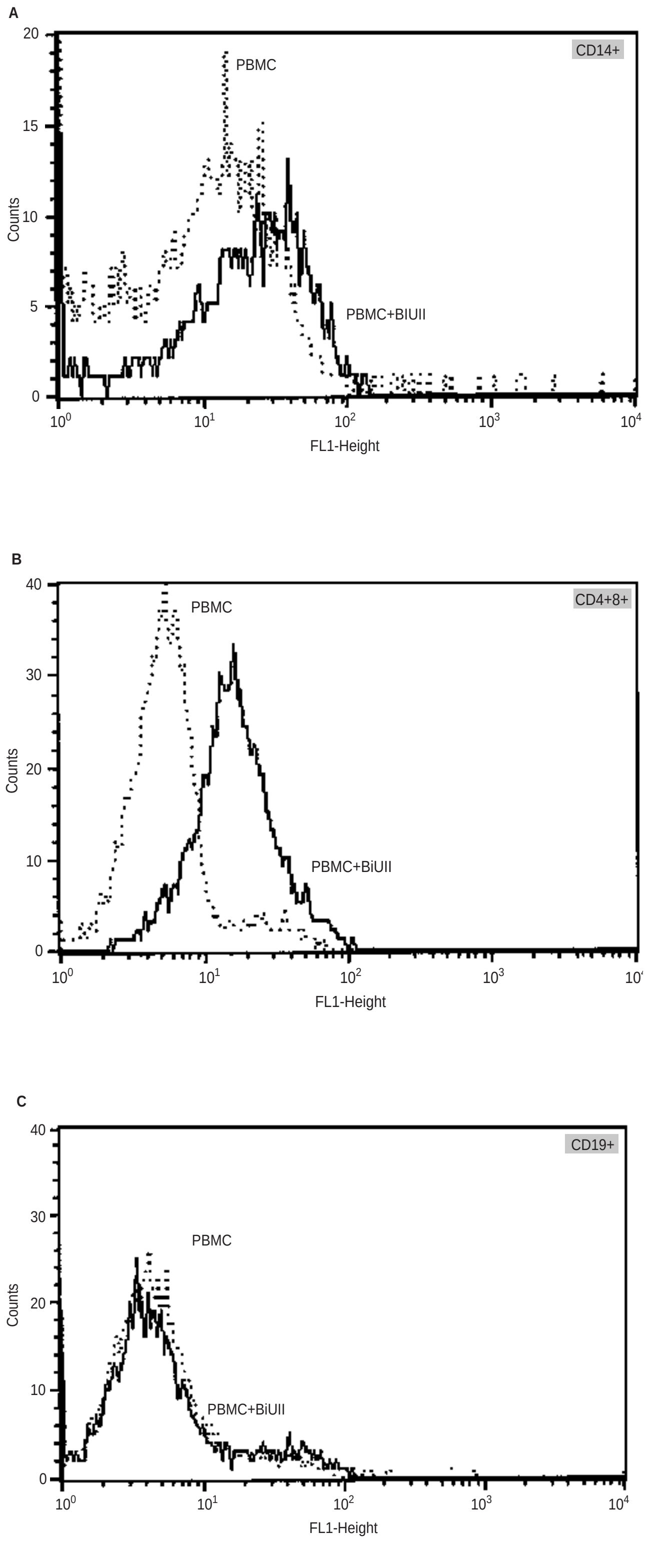

**Figure 1** Binding of BiUII to PBMC subpopulations. PBMCs were incubated with BiUII and binding was assessed by FACS analysis. (**A**) BiUII recognizes, albeit weakly, CD14+ monocytes (black line). As monocytes do not express CD3, binding of BiUII is probably mediated by the Fc-region that binds to FcγRI with high affinity. (**B**) BiUII strongly binds to CD3-expressing CD4+/CD8+ T lymphocytes. (**C**) No binding of BiUII antibodies was observed on CD19+ B cells. Isotype control = dotted line.

cytometry (not shown). Peripheral blood mononuclear cells (PBMC) were isolated from heparinized blood of voluntary donors by Ficoll density centrifugation.

### Monoclonal antibodies mAbs for FACS analysis were from Pharmingen (Hamburg, Germany) except the DC-specific antibody BMA-X11 (Dianova, Hamburg, Germany).

### Generation of dendritic cells

The adherent fraction of PBMCs was incubated for 7 days in Iscove's medium with 5% FCS (both Gibco BRL, Gaithersburg, MD, USA) and 800 U $ml^{-1}$ each of human IL-4 and GM-CSF (both Boehringer Mannheim, Penzberg, Germany).

### FACS® analysis

For FACS® analysis, $10^5$ cells were incubated with the primary antibody for 30 min on ice in PBS with 2% FCS. Cells were washed twice in PBS and incubated for another 30 min with the second, FITC-labelled, antibody. After two final washings, propidiumiodide was added and flow cytometry was performed using a FACSCalibur® cytometer and the CellQuest analysis program (Becton Dickinson, Heidelberg, Germany). For isolation of highly purified $CD2^+$ cells, PBMCs were incubated with FITC-labeled antibodies and separated on a FACS-Calibur®.

### Production of BiUII

The BiUII Quadroma was produced as previously described (Lindhofer et al, 1995). The following hybridomas have been used: 26II6 (rat IgG2b, anti-CD3, provided by R Schuh, GSF, Germany) and C215 (mouse IgG2a, anti-EpCAM, kindly provided by M Dohlsten, Pharmacia Upjohn, Sweden). To isolate hybrid Ab molecules of the subclass combination rat IgG2b/mouse IgG2a from quadroma, the supernatants were centrifuged, filtered, and loaded onto a 5 ml Econo Pac protein A column (Biorad, Richmond, CA, USA). After washing with 10 volumes of PBS, antibodies with the hybrid heavy-chain configuration were eluted with 0.1 M citric acid, pH 5.1.

### Cell culture and killing efficiency

For determination of BiUII-mediated killing of tumour cells and cytokine production, $1 \times 10^4$ PCl-1 cells per well (targets = T) were pipetted in 96-well flat-bottom plates (Falcon) and PBMCs or subpopulations of these effectors (= E) were added at E:T ratios from 40:1 to 1:1. BiUII was used at 10 ng per well in a total volume of 100 µl per well RPMI with 10% FCS. Plates were incubated for 3 days at 37°C in a humified atmosphere and 5% $CO_2$.

### Isolation of monocytes/macrophages and NK cells

CD14+ monocytes/macrophages and CD56+/CD3– NK cells were isolated from PBMCs using PE-labelled monoclonal antibodies and a Becton Dickinson FACSVantage cell sorter. Purity of isolated cells was examined by flow cytometry.

 *© 2000 Cancer Research Campaign*

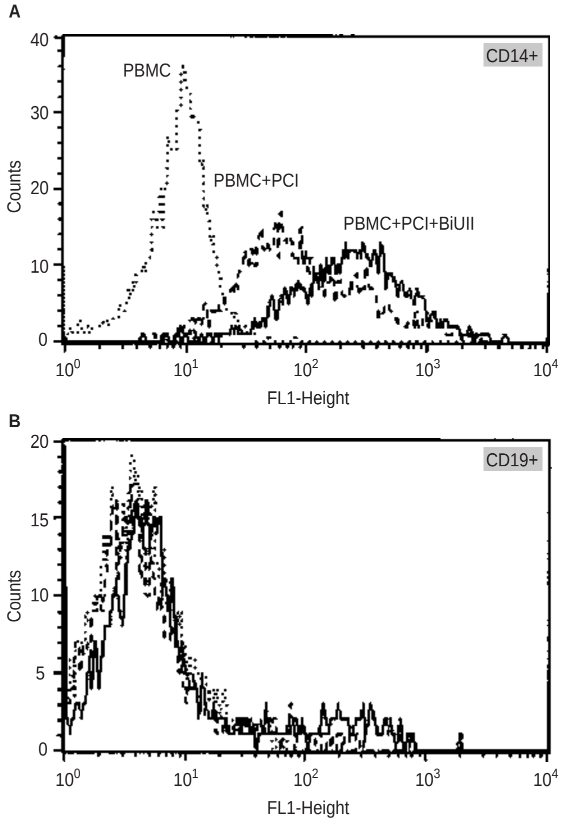

**Figure 2** BiUII stimulates accessory cells to phagocytose PCl tumour cells. FITC-labelled PCl cells were cocultivated with PBMCs for 15 h with or without BiUII. After 15 h, monocytes (CD14+) and B cells (CD19+) were analysed for the presence of FITC, indicating the phagocytosis of PCl cells. (**A**) The most prominent FITC mean values (248) were observed in monocytes incubated in the presence of BiUII. A much lower intensity (76) was observed where BiUII was omitted. Background value of PBMC was 9. Similar results were observed after 24 or 48 h cocultivation period (data not shown). (**B**) In contrast, no FITC was incorporated in B cells.

### FITC-labelling and uptake of PCl-1 tumour cells

PCl-1 cells were washed twice with $Ca^{2+}$ and $Mg^{2+}$ free PBS. 1 µl of FITC (1 mg $ml^{-1}$ in Ethanol; Sigma, Deisenhofen, Germany) was then added to each $2 \times 10^5$ tumour cells in 100 µl PBS, and cells were shaken for 30 min at room temperature. Thereafter, FITC-labelled PCI-1 cells were washed twice with cell culture medium and added to PBMC cultures. The intensity of FITC-labeling was monitored by FACS analysis. Phagocytic capacity of PBMC co-incubated with FITC-labelled PCl-1 cells and BiUII was revealed by FACS after staining with PE-labelled mouse-anti-human-CD14 or –CD19 antibodies. FITC fluorescence intensity of vital CD14+ or CD19+ PBMCs was measured and interpreted as uptake of FITC-labelled PCl-1 tumour cells. Binding of BiUII to PBMC subclasses was revealed by FACS analysis after double-staining with FITC-labelled mouse-anti-rat antibodies (Dianova, Hamburg, Germany) and PE-labelled mouse-anti-human–CD4/CD8, –CD14, or –CD19 antibodies. A combination of gates (vital cells, CD14+ or CD19+ and FCS vs SSC) was used to exclude aggregates of PCl-1 cells with PBMCs from our analysis of phagocytosis of tumour cells.

### MTT-Assay

To assess BiUII-mediated tumour cell killing, a colourimetric MTT-based assay was performed as previously described (Heo et al, 1990). Briefly, PCI-1 target cells were plated in wells of a 96-well flat-bottom plate and incubated overnight to prepare semiconfluent cell monolayers. Effector cells were added to the tumour cell monolayers at the appropriate ratios and plates were incubated for 24–48 h. After removing effectors by washing, MTT solution (0.5 mg $ml^{-1}$; Sigma) was added, and plates were incubated for a further 4 h. The MTT solution was removed and blue crystals of formazan formed in viable tumour cells were dissolved by adding dimethylsulphoxide. Plates were read at 540 nm in a spectrophotometer and results were calculated based on the mean absorbance obtained from at least six wells according to the following formula: % cell death = $100 \times (C–E)/(C–B)$, where C is the optical density reading of the cells with target cells in the absence of effectors (control), B is background without any cell population, and E is the optical density reading of adherent tumour cells remaining in the well after co-incubation with effector cells.

### Activity of GTP cyclohydrolase I and cellular pterin levels

The activity of GTP cyclohydrolase I was determined in the supernatant fraction of the cell extracts (Tris/HCl, pH 8.0; 2.5 mM EDTA) after acidic iodine oxidation of the reaction product dihydroneopterin triphosphate. The neopterin phosphates were separated by ionpairing HPLC and fluorometrically detected. Cellular neopterin and biopterin were determined in aliquots of the cell extracts after acidic iodine oxidation, deproteinization by trichloroacetic acid, pre-purification by cation-exchange chromatography and separation by reverse-phase HPLC, basically as described previously (Kerler et al, 1990).

### RESULTS

### BiUII binds to CD3–, FcγR+ accessory cells

We constructed a new class of bispecific antibody, BiUII, that recognizes epithelial tumour cells via the pan-carcinoma antigen EpCAM (Quak et al, 1990) and redirects T lymphocytes via CD3. We have recently shown that BiUII displays an excellent antitumour activity and also that complete PBMCs are superior to a highly purified T-cell population of the same donor with regard to tumour cell killing (Zeidler et al, 1999). We therefore addressed the question whether BiUIl binds peripheral blood monocytes which express the high-affinity Fcγ-RI, CD64 and whether these accessory cells contribute to tumour cell killing. To this end, PBMCs were incubated with BiUII and a FITC-labelled anti-rat lg antibody and binding was assessed by FACS analysis. As depicted in Figure 1, BiUII binds to CD14+, albeit weakly. Since neither antigen recognized by BiUIl (CD3 and EpCAM) is present on monocytes, we concluded that binding of BiUIl to Fcγ-R-positive accessory cells is most probably mediated by the Fc region of BiUIl. This finding is in agreement with data already published (Haagen et al, 1995). In parallel, we investigated the binding of BiUIl to T and B lymphocytes. T lymphocytes express CD3, one

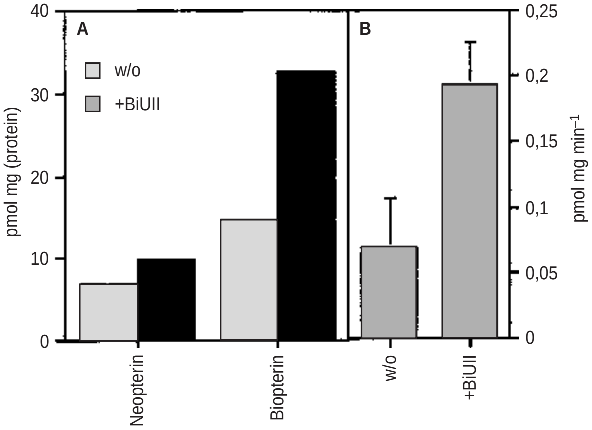

**Figure 3** BiUII induces GTP-cyclohydrolase I activity and the production of neopterin and biopterin in monocytes/macrophages. PCl-1 cells were cocultivated with PBMCs either with (100 ng ml$^{-1}$) or without BiUII for 2 days. (**A**) The production of neopterin and biopterin and (**B**) the activity of GTP cyclohydrolase was determined. BiUII stimulates the production of both biopterin and the monocyte-specific neopterin, as well as the activity of the enzyme. PCl-1 cells per se are negative for all three products.

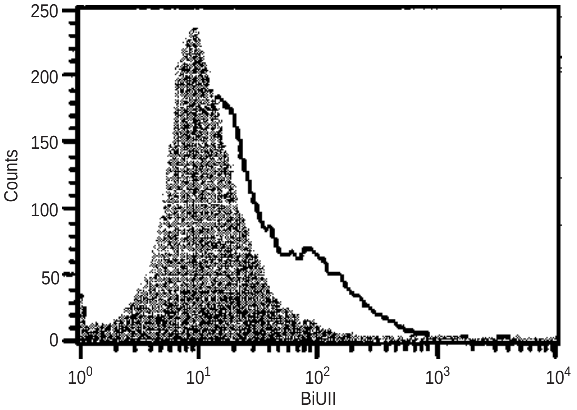

**Figure 4** BiUII binds to NK cells. Binding of BiUII to highly purified, CD16+/CD3-NK cells from peripheral blood was investigated by FACS. Mean values are 64 with (black line) and 24 without BiUII (grey trace).

target molecule for BiUIl, and consequently a strong binding was observed. In contrast, BiUIl does not bind to CD19+ B cells that only express the low-affinity FcγRII, CD32.

### BiUII-mediated phagocytosis of PCl-1 cells by CD14+ monocytes/macrophages

Since accessory cells contribute to T-cell activation and tumour-cell elimination in different ways, we wanted to find out whether direct phagocytosis of the tumour cells by CD14+ cells occurs. To this end, PCI-1 tumour cells were stained with FITC and cocultivated for 15 h with PBMCs in the presence of BiUII to assess direct phagocytosis of tumour cells. In control settings, BiUII and/or FITC-labelled tumour cells were omitted. After concultivation, the mean FITC-fluorescence intensity, indicative for the uptake of labeled PCI-1 cells, was measured in vital CD14+ monocytes or CD19+ B lymphocytes. As shown in Figure 2A, uptake of FITC-fluorescence was triggered in CD14+ monocytes/macrophages co-cultivated with BiUII. In contrast, CD19+ B cells from the same donor showed no signs for PCI-1 uptake, even in the presence of the bispecific molecule (Fig. 2B).

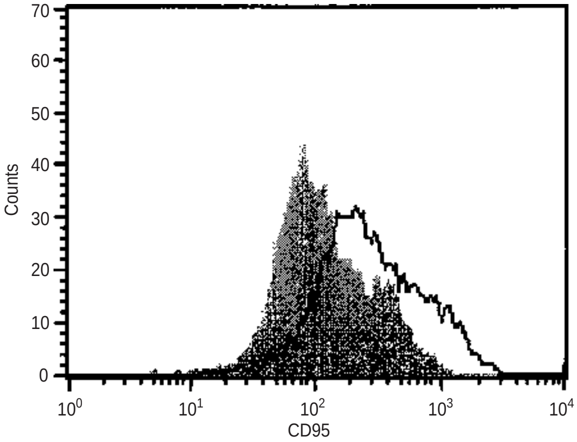

**Figure 5** Addition of BiUII to a culture of purified NK cells induces the expression of CD95, an activation marker for NK cells after 1 day of culture. Mean values are 400 (black line) and 182 (grey trace) for NK cells incubated with and without BiUII, respectively.

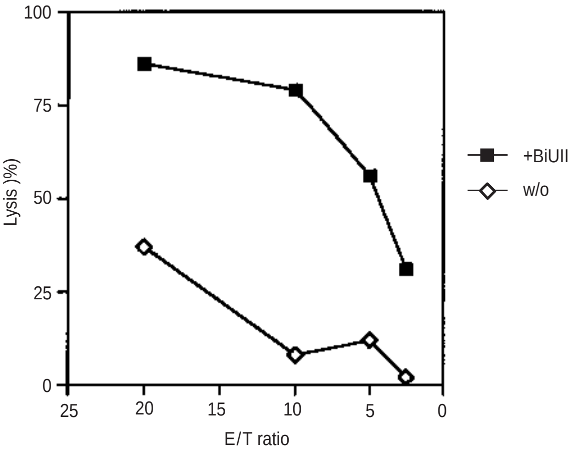

**Figure 6** NK cells are stimulated to kill allogeneic tumour cells after incubation with BiUII. Highly purified CD56+CD3−NK cells (effectors) were cocultivated with PCl-1 cells at different E/T ratios for 2 days either with (100 ng ml$^{-1}$) or without BiUII. Killing of tumour cells (targets) was calculated in a MTT assay as described in Material and methods. One representative of three independent experiments is shown.

### BiUII stimulates the production of neopterin and biopterin

Stimulation of T cells causes release of interferon-γ, which in turn induces increased expression of GTP cyclohydrolase I in monocytes/macrophages and in the T cells themselves (Schott et al, 1993; Ziegler, 1990). This enzyme initiates and controls the biopterin synthesis pathway. Therefore, activated T cells produce tetrahydrobiopterin, whereas monocytes/macrophages cannot complete the pathway. They terminate the synthesis pathway after the first step and instead accumulate and shed neopterin. Increase in the activity of GTP cyclohydrolase I and the synthesis of neopterin and biopterin are therefore indicators of monocyte/macrophage and of T-cell activation, respectively (Ziegler, 1990). Figure 3 demonstrates that BiUII induces GTP cyclohydrolase activity and enhancement of neopterin and biopterin production in PBMC after cocultivation with tumour cells in the presence of BiUII, indicative for activation of T cells and monocytes.

© 2000 Cancer Research Campaign

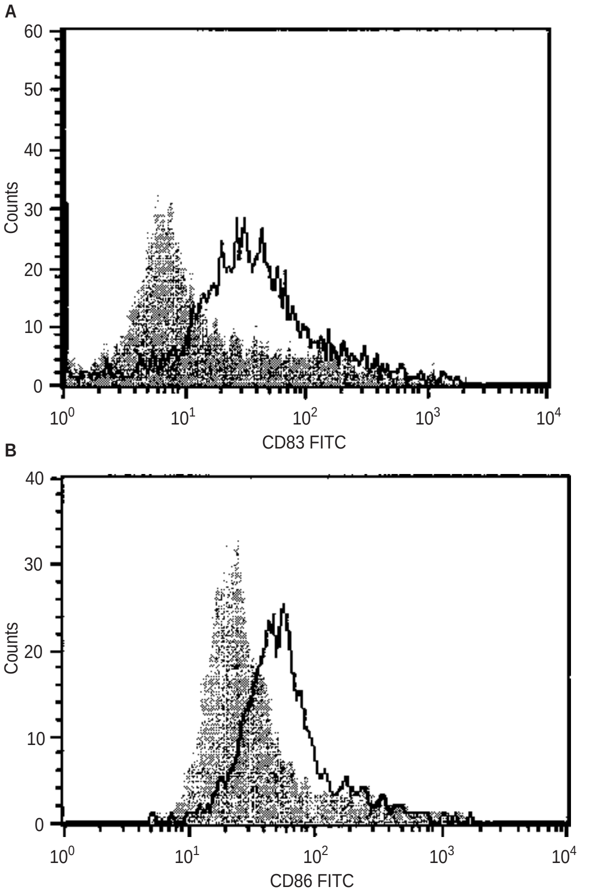

**Figure 7** Induction of CD83 and CD86 on dendritic cells. Addition of BiUIl for 1 day to a culture of DCs leads to upregulation of (**A**) the activation marker CD83 (**B**) and the costimulatory molecule CD86. Only cells positive for the DC marker BMA-X11 were considered for analysis. With BiUIl = black line; without antibody = grey trace.

### BiUIl activates NK cells to tumour cell lysis

NK cells are known to play a pivotal role for the elimination of tumour cells. Since NK cells express the low-affinity FcγRIII (CD16), we wondered whether BiUIl not only binds to CD64+ monocytes/macrophages but also to CD16+ NK cells. We therefore isolated highly purified CD56+/CD3– NK cells and incubated them with BiUIl and revealed binding of the bispecific antibody by FACS analysis (Figure 4).

Binding of BiUII to NK cells via CD16 should lead to their activation, resulting in an antitumour activity. We therefore looked for BiUII-mediated induction of CD95 on NK cells, which is recognized as an activation marker for these cells (Medvedev et al, 1997; Robertson et al, 1995) and investigated tumour-cell killing by BiUII-activated NK cells. As shown in Figure 5, addition of BiUIl to the cell culture induces the expression of CD95 on CD3–/CD16+NK cells indicating their activation via the Fc-region of BiUII. Consequently, cocultivation of NK cells with allogeneic PCI-1 tumour cells in the presence of BiUII resulted in enhanced tumour-cell killing (Figure 6). We observed that NK cells per se display a remarkable activity against allogeneic cells. However, this cytotoxicity was further enhanced by the addition of BiUII.

### BiUIl induces the upregulation of costimulatory molecules on dendritic cells

The network of dendritic cells (DCs) is another class of key regulators of immune responses. DCs are potent antigen-presenting cells (Steinman, 1991) and trigger the activation of T cells, e.g. via the CD40-dependent pathway (McLellan et al, 1996). Activation of DCs is characterized by the neoexpression of CD83 (Czerniecki et al, 1997; Zhou and Tedder, 1996) and upregulation of costimulatory molecules (Cella et al, 1996). Thus, DCs are thought to be involved in the generation of cytotoxic T cells (Cella et al, 1996; Ridge et al, 1998).

The objective of this study was to investigate whether DCs are activated by BiUIl. DCs were generated from the adherent fraction of PBMCs by incubating these cells for 2 weeks in the presence of IL-4 and GM-CSF. The percentage of DCs in the culture was checked by staining with the DC-specific antibody BMA-X11 and was shown to be > 80% (not shown). The DCs were incubated overnight either with BiUII (100 ng $ml^{-1}$) or left untreated in cell culture medium only. After 16 h, the expression of surface markers CD83 and CD86 was revealed by FACS analysis. As shown in Figure 7, incubation of DCs in the presence of BiUII leads to the upregulation of both CD83 and the costimulatory signal CD86, indicating the activation of DCs mediated by our bispecific molecule.

### DISCUSSION

We demonstrate here that not only the two specific binding arms but also the Fc-region of a bispecific antibody can contribute to activation of immune effector cells and thus to anti-tumour activity. However, binding of Fcγ receptors and activation of Fcγ-R expressing cells strictly depends on the composition of the Fc-region of the bispecific molecule. Mouse IgG2a and rat IgG2b are two evolutionally related potent effector subclasses that, in combination, exert efficient activation of human accessory cells. This is shown by:

- the upregulation of costimulatory molecules and activation markers like CD83, CD86, and CD95
- the upregulation of neopterin synthesis
- the direct phagocytosis of tumour cells by purified monocytes, and
- the direct killing by isolated accessory cells without the contribution of T cells.

Interestingly, PBMCs were only weakly activated by equimolar amounts of the two parental monoclonal antibodies (Zeidler et al, 1999).

Conventional bsAbs are usually composed of one potent subclass like mouse IgG2a or rat IgG2b and a less potent subclass like mouse 1gG1 (de Gast et al, 1995), or even two less potent subclasses (Weiner et al, 1993). As a consequence, the Fc-region of conventional bsAbs is usually not able to activate human accessory cells. Instead, these bispecific molecules bind and activate a single class of effector cell via one of their binding arms. This has the drawback that, in the case of T cells, an isolated activation via the CD3 molecule without appropriate costimulatory signals may cause activation-induced anergy (Daniel et al, 1998). We therefore constructed a bispecific antibody that activates more than one class of immune cell, a situation that much more resembles inflammatory and immune reactions in vivo. We have already shown the potential of such new bsAbs in tumour eradication in an animal

*© 2000 Cancer Research Campaign* 

model (Lindhofer et al, 1996). The aim of the experiments presented here was to reveal the mechanisms that are induced by this new agent, in more detail. Therefore, we demonstrate the activation of accessory cells that either express Fcγ-RI (monocytes/macrophages and DCs) or Fcγ-RIII (NK cells). We also show that, for example, monocytes/macrophages not only are activated but also directly contribute to the anti-tumour activity of BiUII by phagocytosis. In contrast, this mouse IgG2a × rat IgG2b bispecific molecule does not bind to B lymphocytes that express the low-affinity receptor CD32. Enhanced production of tetrahydrobiopterin after BiUII stimulation may also participate in the modulation of cell functions, e.g. by increasing NO production (Mayer and Hemmens, 1997). Further, accessory cells deliver molecules like CD40, CD80, and CD86, important for T-cell activation (McLellan et al, 1996; Van Gool et al, 1996) and produce pro-inflammatory cytokines (Zeidler et al, 1999). The significance of accessory-cell activation is underlined by data recently published that demonstrates the importance of CD28 costimulation for the prevention of activation-induced T-cell death in an bsAb-immunotherapy trial (Daniel et al, 1998).

Probably most important, due to the recruitment of different immune effector cells, BiUII-mediated immune complexes represent a self-supporting system that is not dependent on the addition of exogenous IL-2, a fact that is extremely advantageous for in vivo applications. The concerted activation of T cells and accessory cells at the tumour site, leading to the phagocytosis, processing, and presentation of tumour material, may account for the potential of this new class of intact bispecific antibody and is a prerequisite for a polyclonal humoral and cellular immune response. Although HAMA or HARA reactions in vivo cannot be excluded, especially after repeated applications, this new class of bispecific molecule may represent a promising tool for the adjuvant treatment of cancer patients.

### ACKNOWLEDGEMENTS

This work was supported by the Else Kröner-Fresenius-Stiftung, the Rudolf Bartling-Stiftung, and by institutional grants.

### REFERENCES

Cella M, Scheidegger D, Palmer Lehmann K, Lane P, Lanzavecchia A and Alber G (1996) Ligation of CD40 on dendritic cells triggers production of high levels of interleukin-12 and enhances T cell stimulatory capacity: T-T help via APC activation. *J Exp Med* **184**: 747–752

Clynes R, Tekechi Y, Moroi Y, Houghton A and Ravetch JV (1998) Fc receptors are required in passive and active immunity to melanona. *Proc Natl Acad Sci USA* **95**: 652–656

Czerniecki BJ, Carter C, Rivoltini L, Koski GK, Kim HI, Weng DE, Roros JG, Hijazi YM, Xu S, Rosenberg SA and Cohen PA (1997) Calcium ionophore-treated peripheral blood monocytes and dendritic cells rapidly display characteristics of activated dendritic cells. *J Immunol* **159**: 3823–3837

Daniel PT, Kroidl A, Kopp J, Sturm I, Moldenhauer G, Dörken B and Pezzutto A (1998) Immunotherapy of B-cell lymphoma with CD3×19 bispecific antibodies: costimulation via CD28 prevents 'veto' apoptosis of antibody-targeted cytotoxic T cells. *Blood* **92**: 4750–4757

de Gast GC, Haagen IA, van Houten AA, Klein SC, Duits AJ, de Weger RA, Vroom TM, Clark MR, Phillips J and van Dijk AJ (1995) CD8 T cell activation after intravenous administration of CD3 × CD19 bispecific antibody in patients with non-Hodgkin lymphoma. *Cancer Immunol Immunother* **40**: 390–396.

Fanger MW, Segal DM and Wunderlich JR (1990) Going both ways: bispecific antibodies and targeted cellular cytotoxicity. *FASEB J* **4**: 2846–2850

Haagen IA, Geerars AJ, Clark MR and van de Winkel JG (1995) Interaction of human monocyte Fc gamma receptors with rat lgG2b. A new indicator for the Fc gamma Rlla (R-H131) polymorphism. *J Immunol* **154**: 1852–1860

Heo DS, Park JG, Hata K, Day R, Herberman RB and Whiteside TL (1990) Evaluation of tetrazolium-based semiautomatic colorimetric assay for measurement of human antitumour cytotoxicity. *Cancer Res* **50**: 3681–3690

Inaba K and Steinman RM (1984) Resting and sensitized T lymphocytes exhibit distinct stimulatory (antigen-presenting cell) requirements for growth and lymphokine release. *J Exp Med* **160**: 1717

Kerler F, Hültner L, Ziegler I, Katzenmaier G and Bacher A (1990) Analysis of the tetrahydrobiopterin synthesizing system during maturation of murine reticulocytes. *J Cell Physiol* **142**: 268–271

Klein SC, Boer LH, De Weger RA, De Gast GC and Bast EJEG (1997) Release of cytokines and soluble cell surface molecules by PBMC after activation with the bispecific antibody CD3×CD19. *Scand J Immunol* **46**: 452–458

Lindhofer H, Menzel H, Günther W, Hültner L and Thierfelder S (1996) Bispecific antibodies target operationally tumourspecific antigens in two leukemia relapse models. *Blood* **88**: 4651.

Lindhofer H, Mocikat R, Steipe B and Thierfelder S (1995) Preferential species-restricted heavy/light chain pairing in rat/mouse quadromas. Implications for a single-step purification of bispecific antibodies. *J Immunol* **155**: 219–225

Mayer B and Hemmens B (1997) Biosynthesis and action of nitric oxide in mammalian cells. *Trends Biochem Sci* **22**: 477–481

McLellan AD, Sorg RV, Williams LA and Hart DN (1996) Human dendritic cells activate T lymphocytes via a CD40:CD40 ligand-dependent pathway. *Eur J Immunol* **26**: 1204–1210

Medvedev AE, Johnsen AC, Haux J, Steinkjer B, Egeberg K, Lynch DH, Sundan A and Espevik T (1997) Regulation of Fas and Fas-ligand expression in NK cells by cytokines and the involvement of Fas-ligand in NK/LAK cell-mediated cytotoxicity. *Cytokines* **9**: 394–404

Quak JJ, Van Dongen G, Brakkee JG, Hayashida DJ, Balm AJ, Snow GB and Meijer CJ (1990) Production of a monoclonal antibody (K 931) to a squamous cell carcinoma associated antigen identified as the 17–1A antigen. *Hybridoma* **9**: 377–387

Ridge JP, Di Rosa, F and Matzinger P (1998) A conditioned dendritic cell can be a temporal bridge between a CD4+ T-helper and a T-killer cell. *Nature* **393**: 474–478

Robertson MJ, Manley TJ, Pichert G, Cameron C, Cochran KJ, Levine H and Ritz J (1995) Functional consequences of APO-1/Fas (CD95) antigen expression by normal and neoplastic hematopoietic cells. *Leuk Lymphoma* **17**: 51–61

Schott K, Guetlich M and Ziegler I (1993) Induction of GTP-cyclohydrolase I mRNA expression by lectin activation and interferon-gamma treatment in human cells associated with the immune response. *J Cell Physiol* **56**: 12–16

Steinman RM (1991) The dendritic cell system and its role in immunogenicity. *Annu Rev Immunol* **9**: 271–296

Stüber E, Strober W and Neurath M (1996) Blocking the CD40L-CD40 interaction in vivo specifically prevents the priming of T helper 1 cells through inhibition of interleukin 12 secretion. *J Exp Med* **183**: 693

Valerius T, Stockmeyer B, vam Spriel A, Graziano R, van den Herik-Oudijk I, Repp R, Deo Y, Lund J, Kalden J, Gramatzki M and van de Winkel JGJ (1997) FcαRI (CD89) as a novel trigger molecule for bispecific antibody therapy. *Blood* **90**: 4485–4492

Van Gool SW, Vandenberghe P, de Boer M and Ceuppens JL (1996) CD80, CD86 and CD40 provide accessory signals in a multiple-step T-cell activation model. *Immunol Rev* **153**: 47–83

Weiner LM, Clark JI, Ring DB and Alpaugh RK (1995) Clinical development of 2B1, a bispecific murine monoclonal antibody targeting c-erbB-2 and Fc gamma RIII. *J Hematother* **4**: 453–456

Weiner LM, Holmes M, Adams GP, LaCreta F, Watts P and Garcia de Palazzo I. (1993) A human tumour xenograft model of therapy with a bispecific monoclonal antibody targeting c-erbB-2 and CD16. *Cancer Res* **53**: 94–100

Zeidler R, Reisbach G, Lang S, Wollenberg B, Chaubal S, Schmitt B and Lindhofer H (1999) Simultaneous activation of T cells and accessory cells by a new class of intact bispecific antibody results in efficient tumour cell killing. *J Immunol* **163**: 1246–1252

Zhou LJ and Tedder TF (1996) CD14+ blood monocytes can differentiate into functionally mature CD83+ dendritic cells. *Proc Natl Acad Sci USA* **93**: 2588–2592

Ziegler I (1990) Production of pteridines during hematopoiesis and T-lymphocyte proliferation: Potential participation in the control of cytokine signal transmission. *Med Res Rev* **10**: 95–114

© 2000 Cancer Research Campaign
